# A Method of Calculating Motion Error in a Linear Motion Bearing Stage

**DOI:** 10.1155/2015/696417

**Published:** 2015-01-29

**Authors:** Gyungho Khim, Chun Hong Park, Jeong Seok Oh

**Affiliations:** Department of Ultra Precision Machines and Systems, Korea Institute of Machinery and Materials, 156 Gajeongbuk-ro, Yuseong-gu, Daejeon 305-343, Republic of Korea

## Abstract

We report a method of calculating the motion error of a linear motion bearing stage. The transfer function method, which exploits reaction forces of individual bearings, is effective for estimating motion errors; however, it requires the rail-form errors. This is not suitable for a linear motion bearing stage because obtaining the rail-form errors is not straightforward. In the method described here, we use the straightness errors of a bearing block to calculate the reaction forces on the bearing block. The reaction forces were compared with those of the transfer function method. Parallelism errors between two rails were considered, and the motion errors of the linear motion bearing stage were measured and compared with the results of the calculations, revealing good agreement.

## 1. Introduction

Motion error in precision stages, which is typically evaluated after fabrication, is caused primarily by profile errors in the guide rails. If the motion error is not within the target accuracy, the guide rails should be modified via a process such as regrinding, scraping, or lapping. This process requires highly skilled workers and is time-consuming and costly. Simulating the motion error at the design stage can enable engineers to understand the effects of the design parameters on motion accuracy, and the allowable tolerance in the rail-form error may be assessed. This can reduce the development time, as well as improve motion accuracy by changing design parameters [1, 2].

The load distributions of linear motion ball guides have been analyzed using the Hertz contact theory, showing that motion errors can be maintained within the magnitude of the Gaussian misalignment of the rail by accuracy averaging effects [3, 4]. The transfer function method has been used to calculate motion errors in two degrees of freedom (DOFs) using a hydrostatic feed table, with a reverse technique to determine the rail-form errors from the measured motion errors [5, 6]. A similar approach has been followed to describe motion error in a linear motion bearing table, and the motion error was reduced to less than a micrometer by correcting for the rail-form error [7]. We have previously extended the transfer function method to analyze motion errors in five DOFs [8]. An analytical model for 5-DOF motion errors in linear motion stages was proposed and experimentally verified for an aerostatic linear motion stage. This method can be applied to various configurations of the stages, as well as different types of bearing. Quite recently, this approach has been used to analyze motion errors in slide table using Turcite pad [9].

Rail-form errors are required to apply the transfer function method. Arbitrary rail-form errors may be assumed for a theoretical analysis; however, for an actual system, they should be measured. Unfortunately, it is not straightforward to obtain the rail-form errors in a linear motion bearing stage, because the surface is composed of several circular-profile grooves. Even if the surfaces of the rails are assumed to be flat, it is not straightforward to measure the rail-form errors. A new method that does not use rail-form errors to estimate motion errors is therefore required.

Here, we described a modification of the transfer function method for application to linear motion bearing stages. The method uses straightness errors of a bearing block rather than rail-form errors to estimate motion errors with five DOFs. The straightness errors of a bearing block can easily be measured by using a laser interferometer or a dial gauge and a straight edge during the assembly process. The remainder of the procedure is as described in [8], except that the reaction forces of the bearings are calculated using the bearing stiffness and the straightness errors of the bearing block. The horizontal straightness errors of the bearing block are compensated for to remove errors caused by roll error. Parallelism errors between two rails are considered when determining the motion errors of the stage. To verify the proposed method, the straightness error of a bearing block is calculated based on the rail-form errors using the Hertz contact equation, and the reaction forces from the proposed method are compared with those of the transfer function method. The 5-DOF motion errors estimated using the method described here are then compared with measurements.

## 2. Modeling Motion Error with 5 DOFs

### 2.1. Reaction Force of a Linear Motion Bearing

**Figure 1:**
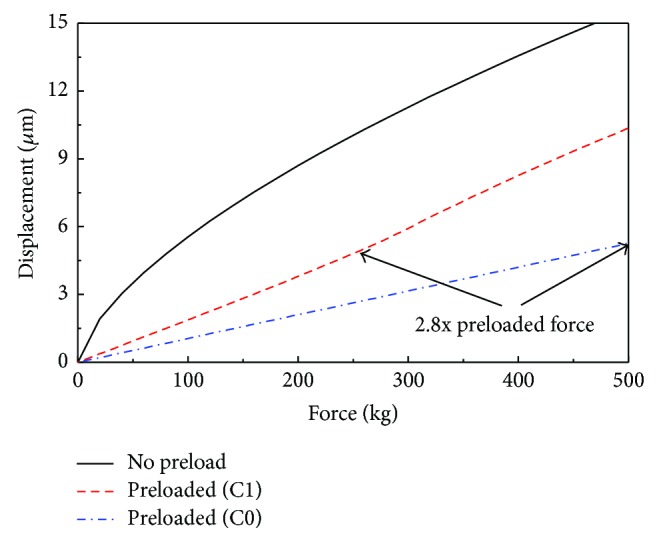
The displacement of a linear motion bearing as a function of the applied force.

Linear motion bearings are used mainly with a preload to achieve a high stiffness. When the preload was applied to the bearing, the displacement of the bearing increased linearly with the applied force, until a force of 2.8-fold the preload force was reached [10], as shown in Figure 1. The bearing stiffness can therefore be considered constant with a preload and when the applied force does not exceed 2.8 times the preload. Even when no preload is applied to the bearing, the stiffness can be assumed to be constant within a small variation of deformation, that is, under normal operating conditions.

**Figure 2:**
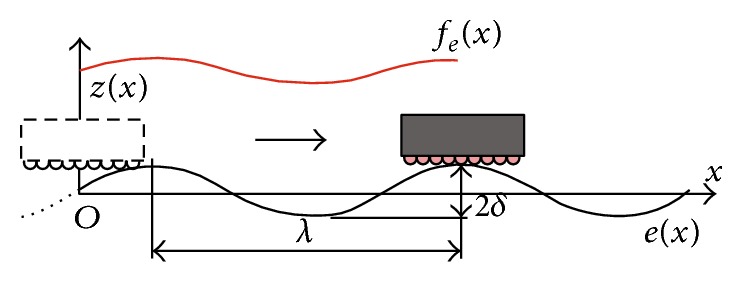
The reaction force of a linear motion bearing.

Figure 2 shows the motion of a linear motion bearing along a rail, with a wavelength *λ* and amplitude *δ*. Here *z*(*x*) is the vertical motion error, and *f*_*e*_(*x*) is the reaction force of the bearing assuming that the motion is linear along the rail and there is no variation in the motion error. The vertical motion error and the reaction force of the bearing have the same wavelength as the rail-form error *e*(*x*), but the magnitude may vary.

The transfer function is introduced to calculate the reaction forces of the bearing [5, 7, 8]. This is defined as the ratio of the amplitude of *f*_*e*_(*ω*) to that of *e*(*ω*) at a spatial frequency *ω* (where *ω* = 2*π*/*λ*). Therefore, the reaction force is calculated from the rail-form error multiplied by the transfer function *K*(*ω*); that is,

(1) $$\begin{array}{c} f_{e}\left(x\right)=\overset{\infty }{\underset{k=0}{\sum }}K\left(\omega_{k}\right)e_{k}\left(x\right),\\ e_{k}\left(x\right)=a_{k}\cos\frac{2k\pi }{L}x+b_{k}\sin\frac{2k\pi }{L}x,\\ e\left(x\right)=\overset{\infty }{\underset{k=0}{\sum }}e_{k}\left(x\right), \end{array}$$

where *L* is the length of the rail; *a*_*k*_ and *b*_*k*_ are the* k*th Fourier cosine and sine coefficients of *e*(*x*), respectively; and *e*_*k*_(*x*) is a rail-form error caused by the Fourier coefficients of *a*_*k*_ and *b*_*k*_.

The rail-form errors are required to calculate motion errors, and the reaction forces can be determined using (1). However, obtaining the rail-form errors for a linear motion bearing stage is not straightforward because of the circular-profile surfaces on the rails. An alternative method is to calculate the reaction force directly from the vertical motion error of the bearing, without using the transfer function and the rail-form errors. The bearing stiffness *K*_*z*_ is assumed to be constant within a range of rail-form error. Therefore, the reaction force of the bearing can be expressed as

(2) $$\begin{array}{c} f_{e}\left(x\right)=K_{z}\cdot z\left(x\right). \end{array}$$

The linear motion bearing can be translated on the rail without requiring the entire system. Therefore, the vertical motion error (or straightness error) of the bearing block can easily be measured using a laser interferometer or dial gauge and straight edge.

Equation (2) can be written using the Fourier series of the measured straightness error; that is,

(3) $$\begin{array}{c} f_{e}\left(x\right)=K_{z}\cdot z\left(x\right)=\overset{\infty }{\underset{k=0}{\sum }}K_{z}\left(a_{k}^{\prime }\cos\frac{2k\pi }{L^{\prime }}x+b_{k}^{\prime }\sin\frac{2k\pi }{L^{\prime }}x\right), \end{array}$$

where *a*_*k*_′ and *b*_*k*_′ are the Fourier coefficients of the straightness errors of the bearing block and *L*′ is the length of the measurement of the straightness error.

The transfer function at *ω* = 0 corresponds to a static stiffness of the bearing block so that *K*(0) = *K*_*z*_. The transfer function at other frequencies determines the stiffness corresponding to rail-form errors of that frequency, as well as the accuracy averaging effect at that frequency. Therefore, the straightness error in the bearing block includes these effects after the transfer function is applied to the rail-form errors.

We calculated the reaction forces obtained from the transfer function method and the method described here. First, the transfer function and static stiffness of the bearing block were calculated. The straightness error of the bearing block was then calculated from the assumed rail-form errors. The reaction forces were calculated, and the results using the two methods were compared.

### 2.2. Straightness of a Bearing Block

**Figure 3:**
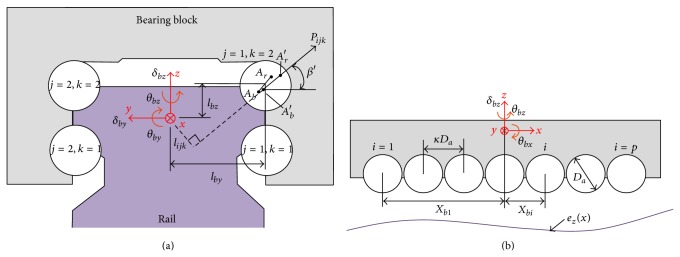
Model of a linear motion bearing block. (a) Front view and (b) side view.

**Figure 4:**
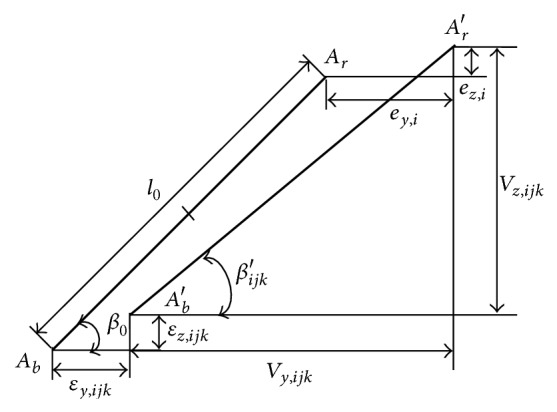
Diagram showing the calculation of the deformation of the ball with *j* = 1 and *k* = 2.

The motion error of a bearing block depends on the deformation of the balls caused by the rail-form errors as well as bed errors onto which the rails are bolted. In this paper, we assume the rail-form errors include the bed errors. Figures 3 and 4 show the model of the bearing block. Here *A*_*r*_ and *A*_*r*_′ are the ideal and actual centers of curvature of the rail grooves; *A*_*b*_ and *A*_*b*_′ are the ideal and actual centers of the bearing block grooves; *β*_0_ and *β*′ are the ideal and actual contact angles of the balls; and *i*, *j*, and *k* are the indices of the balls in the *x*-, *y*-, and *z*-axis, respectively. The linear motion bearing has four components of rail-form errors corresponding to each of the grooves; however, they can be assumed to be the same in the vertical and horizontal directions.

Let *δ*_*bz*_ and *δ*_*by*_ be the vertical and horizontal straightness errors, and *θ*_*bx*_, *θ*_*by*_ and *θ*_*bz*_ the roll, pitch, and yaw errors at the center of the bearing block. The center of the bearing block groove, *A*_*b*_, corresponding to the* i*,* j,* and* k*th ball, moves with a vertical displacement *ε*_*z*,*ijk*_ and horizontal displacement *ε*_*y*,*ijk*_, which can be expressed as follows:

(4) εz,ijkx=δbz(x)−Xbi·θby(x) +−1jlby−l02cos⁡β0·θbxx,εy,ijkx=δbyx+Xbi·θbzx −−1klbz−l02sinβ0·θbxx,

where *X*_*bi*_ is the distance in the *x*-axis between the center of the *i*th ball and the center of the bearing block; *l*_*by*_ and *l*_*bz*_ are the distances between the center of the block and the center of each ball in the *y*- and *z*-axis, respectively; and *l*_0_ is the initial distance between the centers of curvature *A*_*b*_ and *A*_*r*_. We then have

(5) $$\begin{array}{c} X_{bi}=\kappa \cdot D_{a}\left\{i-\frac{p+1}{2}\right\}, \end{array}$$

where *κ* is the interval factor between two balls, and *D*_*a*_ is the diameter of the ball, and *p* is the number of balls in each row.

The center of curvature of the block groove *A*_*b*_ becomes *A*_*b*_′, due to the motion error of the bearing block, and the center of curvature of the rail groove *A*_*r*_ becomes *A*_*r*_′, due to the rail-form errors, as shown in Figure 4. The difference in the distance between the centers of curvature *A*_*b*_ and *A*_*r*_ may then be regarded as the elastic deformation of the ball, *ξ*_*ijk*_, which can be expressed as follows [7, 11]:

(6) $$\begin{array}{c} \xi_{ijk}\left(x\right)=\bar{A_{b}^{\prime }A_{r}^{\prime }}-\bar{A_{b}A_{r}}=\sqrt{V_{y,ijk}{\left(x\right)}^{2}+V_{z,ijk}{\left(x\right)}^{2}}-l_{0}+\chi_{p},\\ l_{0}=2R-D_{a},\\ V_{y,ijk}\left(x\right)=l_{0}\cos\beta -{\left(-1\right)}^{j}\varepsilon_{y,ijk}\left(x\right)+{\left(-1\right)}^{j}e_{y,i}\left(x\right),\\ V_{z,ijk}\left(x\right)=l_{0}\sin\beta -{\left(-1\right)}^{k}\varepsilon_{z,ijk}\left(x\right)+{\left(-1\right)}^{k}e_{z,i}\left(x\right), \end{array}$$

where *χ*_*p*_ is the preload on the ball and *R* is the radius of curvature of the rail and the bearing block grooves.

By applying the Hertz contact equation [7, 11, 12], the force *P*_*ijk*_ that acts on each ball can be obtained; that is,

(7) $$\begin{array}{c} P_{ijk}\left(x\right)=C\sqrt{D_{a}}{\left(\xi_{ijk}\left(x\right)\right)}^{3/2}, \end{array}$$

where *C* is a constant that depends on the ratio of the radius of curvature of the groove to the diameter of the ball.

The equilibrium equations of forces and moments for five-directions on the bearing block can be expressed as follows:

(8) $$\begin{array}{c} \overset{2}{\underset{k}{\sum }}\overset{2}{\underset{j}{\sum }}\overset{p}{\underset{i}{\sum }}\left(P_{ijk}\left(x\right)\times \sin\beta_{ijk}^{\prime }\left(x\right)\right)=0,\\ \overset{2}{\underset{k}{\sum }}\overset{2}{\underset{j}{\sum }}\overset{p}{\underset{i}{\sum }}\left(P_{ijk}\left(x\right)\times \sin\beta_{ijk}^{\prime }\left(x\right)\times X_{bi}\right)=0,\\ \overset{2}{\underset{k}{\sum }}\overset{2}{\underset{j}{\sum }}\overset{p}{\underset{i}{\sum }}\left(P_{ijk}\left(x\right)\times l_{ijk}\left(x\right)\right)=0,\\ \overset{2}{\underset{k}{\sum }}\overset{2}{\underset{j}{\sum }}\overset{p}{\underset{i}{\sum }}\left(P_{ijk}\left(x\right)\times \cos\beta_{ijk}^{\prime }\left(x\right)\right)=0,\\ \overset{2}{\underset{k}{\sum }}\overset{2}{\underset{j}{\sum }}\overset{p}{\underset{i}{\sum }}\left(P_{ijk}\left(x\right)\times \cos\beta_{ijk}^{\prime }\left(x\right)\times X_{bi}\right)=0, \end{array}$$

(9) lijkx =−1j−1k  ·lby−l02cos⁡β0+−1jεy,ijk(x)sinβijk′(x)−lbz−l02sinβ0+−1kεz,ijk(x)cos⁡βijk′(x)βijk′x=tan−1Vz,ijkxVy,ijkx,

where *l*_*ijk*_ is the length of the arm for roll moment, considering the direction of the moment, which can be calculated using the center point coordinates *A*_*b*_′ of the bearing block groove following deformation of the ball.

The motion errors of the bearing blocks *δ*_*bz*_, *δ*_*by*_, *θ*_*bx*_, *θ*_*by*_, and *θ*_*bz*_ can be calculated by solving (8) using a numerical method, such as the Newton-Raphson method. The straightness errors of the bearing block, *δ*_*bz*_ and *δ*_*by*_, are required to calculate the reaction forces of the bearing block using (3).

### 2.3. Comparison of the Reaction Forces

**Figure 5:**
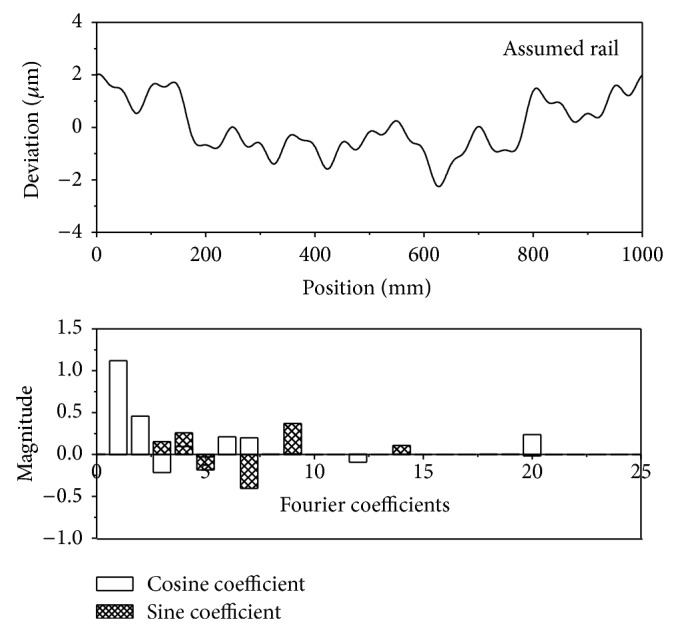
The assumed rail error and Fourier coefficients.

**Figure 6:**
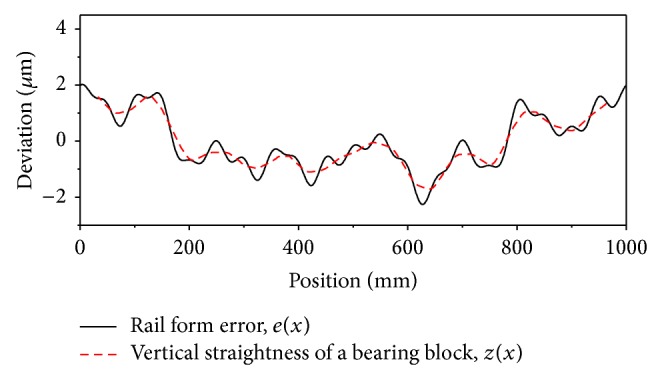
A comparison of the rail-form error and the calculated vertical straightness error.

Figure 5 shows the assumed rail-form errors, together with their Fourier coefficients, which show high-frequency components up to 20.  Figure 6 shows a comparison of assumed rail and vertical straightness errors of the bearing block calculated according to the method described in Section 2.2. The overall agreement is satisfactory; however, the high-spatial-frequency components are clearly lacking. The straightness error is less than the rail-form error because of the averaging effects.

**Figure 7:**
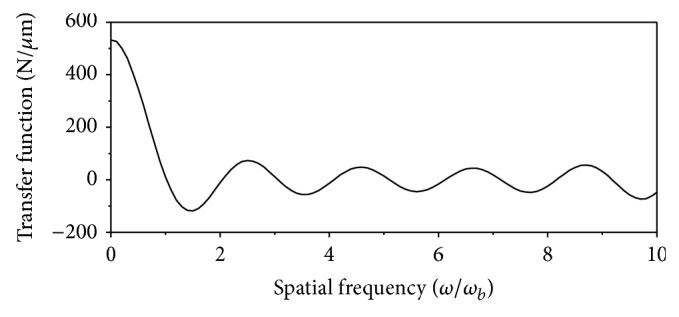
The transfer function in the vertical direction as a function of the spatial frequency.

Figure 7 shows the transfer function in a vertical direction for the bearing block as a function of the spatial frequency. Here a spatial frequency of *ω*/*ω*_*b*_ = 1 is the period of the rail-form error, corresponding to the length of the bearing block. The vertical transfer function was calculated by summing the vertical components of the reaction forces, *P*_*ijk*_, at the spatial frequency *ω* of the rail-form error with a unit magnitude, assuming that the bearing block moves with no motion errors. A detailed explanation of this can be found in [7, 8].

**Figure 8:**
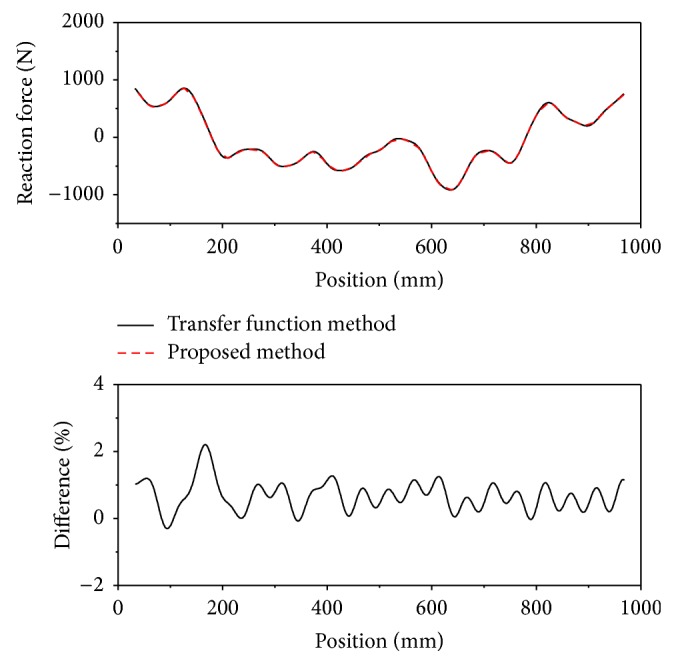
The reaction force calculated using the transfer function method and using the method described here.

Figure 8 shows a comparison of the calculated reaction forces using the transfer function method of (1), and that obtained from (3), where the static stiffness of the bearing block and straightness error were used to calculate the reaction force. The difference between the two was less than 2.2%.

### 2.4. Modeling Motion Error in 5 DOFs

The five components of the motion error in a linear motion bearing stage can be calculated by extending the method described in Section 2.2. However, this method can be cumbersome and furthermore is not flexible for different configurations. In addition, the method requires the rail-form errors. We have previously developed an algorithm based on the transfer function for 5-DOF motion errors, which was applied to an aerostatic bearing stage [8]. This can be applied to an arbitrary type of bearing and was used here to calculate motion errors. The reaction forces of the bearing blocks were calculated using the method described in Section 2.1 for practical application.

**Figure 9:**
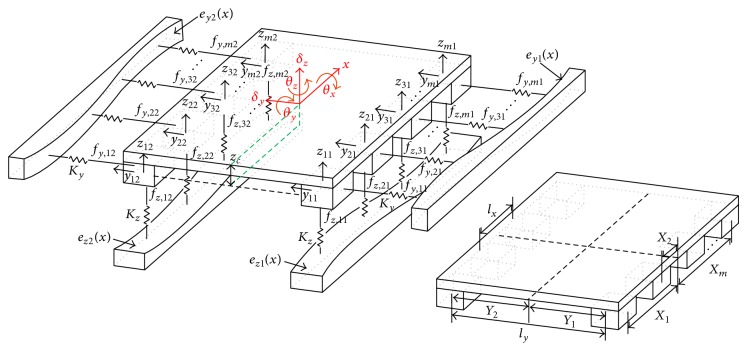
Analytic model for estimating 5-DOF motion errors.

Figure 9 shows the analytical model used to calculate the five components of the motion error of a linear motion bearing stage. The model is similar to the model described in [8]. Therefore, only brief explanations will be given in this section.

The origin of the coordinate system is located on the measurement point of the table center. The vertical displacement *z*_*ij*_ and horizontal displacement *y*_*ij*_ due to the rail-form error in the *i*,*j*th bearing block can be expressed as

(10) $$\begin{array}{c} z_{ij}\left(x\right)=\delta_{z}\left(x\right)-X_{i}\cdot \theta_{y}\left(x\right)+Y_{j}\cdot \theta_{x}\left(x\right),\\ y_{ij}\left(x\right)=\delta_{y}\left(x\right)+X_{i}\cdot \theta_{z}\left(x\right)+z_{c}\cdot \theta_{x}\left(x\right), \end{array}$$

where *z*_*c*_ is the distance between the bearing center and measurement point (the origin of the coordinate system) in the *z* direction; *m* is the number of bearings per rail; *n* is the number of rails; and *X*_*i*_ is the distance between the *i*th bearing center and the table center in the *x* direction, and *Y*_*j*_ is the distance between the *j*th rail center and the table center in the *y* direction, which are given by

(11) $$\begin{array}{c} X_{i}=l_{x}\left\{i-\frac{m+1}{2}\right\},\;\;Y_{j}=l_{y}\left\{j-\frac{n+1}{2}\right\}. \end{array}$$

The equilibrium forces and moments can be expressed as follows:

(12) ∑jn∑imfz,ijx−Kzzijx=0,∑jn∑imfz,ij(x)−Kzzij(x)Xi=0,∑jn∑imfz,ij(x)−Kzzij(x)Yj  +zc∑jn∑imfy,ijx−Kyyijx=0,∑jn∑imfy,ij(x)−Kyyij(x)=0,∑jn∑imfy,ij(x)−Kyyij(x)Xi=0,

where *f*_*z*,*ij*_ and *f*_*y*,*ij*_ are reaction forces that act on the *i*, *j*th bearing in the *z*- and *y*-axis when the bearing moves in a straight line with no variation in the motion error; *K*_*z*_ and *K*_*y*_ are the stiffness of the bearing in the *z*- and *y*-axis, respectively; and *l*_*x*_ and *l*_*y*_ are the distances between two adjacent bearings in the *x*- and *y*-axis, respectively.

The reaction forces *f*_*z*,*ij*_ and *f*_*y*,*ij*_ are calculated using (1) with the rail-form errors, or using (3) with the straightness error from the bearing block. If parallelism errors between two rails exist, they may be regarded as additional rail-form errors of one rail against the other. The reaction forces therefore increase with this parallelism error. The five components of the motion errors are calculated by solving (12).

## 3. Experiment

### 3.1. Straightness Error of the Bearing Block

**Figure 10:**
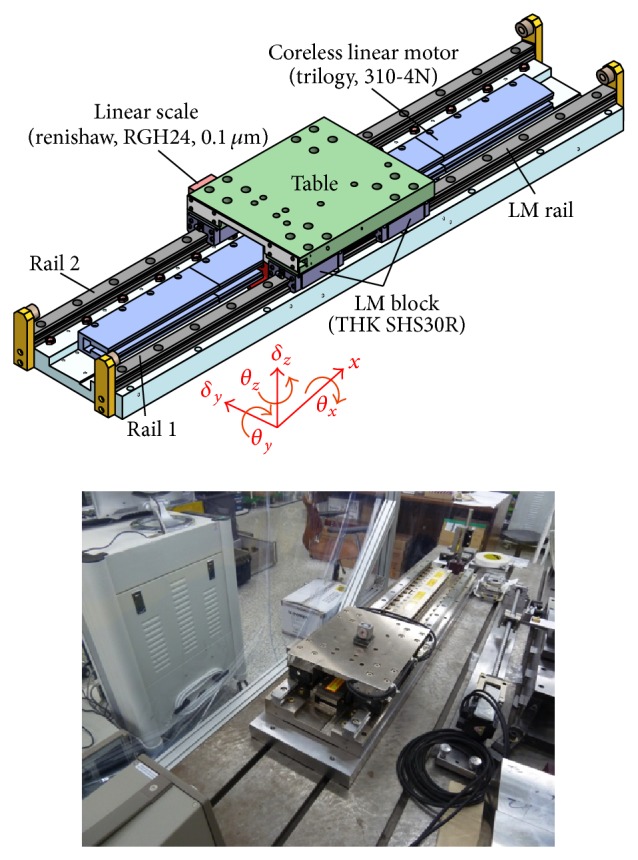
The linear motion bearing stage used in the experiments.

Figure 10 shows the linear motion bearing stage for the experiments. The stage had four linear motion bearings (THK SHS30R), a linear motor (Trilogy 310-4N), and a linear scale (Renishaw, RGH24). The specifications are listed in Table 1.

**Figure 11:**
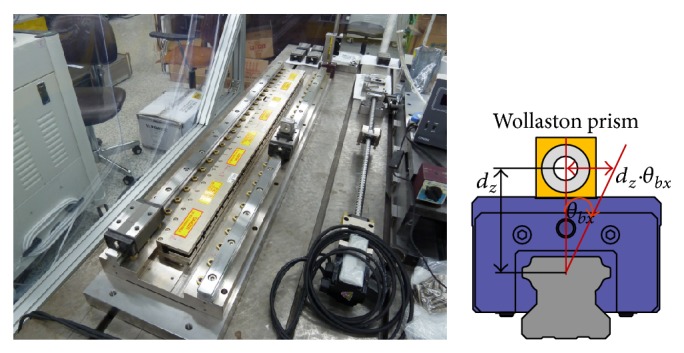
The setup used to measure the straightness error.

The straightness error was measured using a laser interferometer (Agilent 5530) in the vertical and horizontal directions during translation of the bearing block by 10 mm. The horizontal component of the straightness error was influenced by the roll error because the Wollaston prism was installed far from the center of the bearing block in the vertical direction. This resulted in the straightness error *d*_*z*_ · *θ*_*bx*_, as shown in Figure 11, where *d*_*z*_ is the distance between the center of the bearing block and the center of the Wollaston prism in the vertical direction, and *θ*_*bx*_ is the roll error of the bearing block. Therefore, the roll error was measured using an electronic level meter (Mahr 832F).

**Figure 12:**
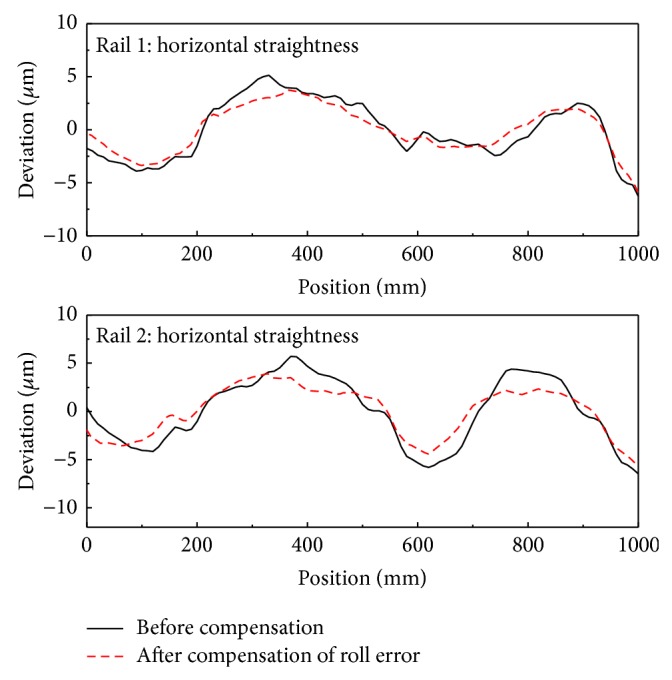
The horizontal component of the straightness error before and after compensating for roll error.

Figure 12 shows the straightness error of the bearing block obtained before and after compensating for roll error. These data were averaged over five measurements.

**Figure 13:**
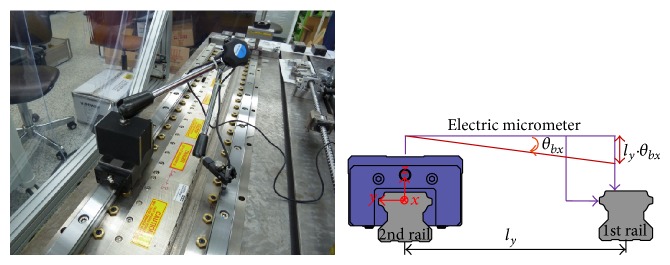
Measurement of the parallelism error between the two rails.

The roll error of the stage is affected by vertical parallelism error between two rails, which was measured with electric micrometers (CITIZEN DTH-P40S) using the setup shown in Figure 13. The micrometers were installed on the bearing block and the rail, and the relative displacement was measured while the bearing block was translated through 10 mm. The parallelism error *l*_*y*_ · *θ*_*bx*_ due to the roll error of the reference bearing block on the second rail was compensated for. The slope of the relative displacement following compensation was regarded as the parallelism error.

Strictly speaking, this was not a parallelism error between the two rails; however, it was sufficient for calculating the roll error of the stage because the straightness error of the bearing block is similar to the rail-form error, except for the high-frequency components, as shown in Figure 6. The parallelism error in the horizontal direction was also measured using the same method, even though it was expected to be negligible.

**Figure 14:**
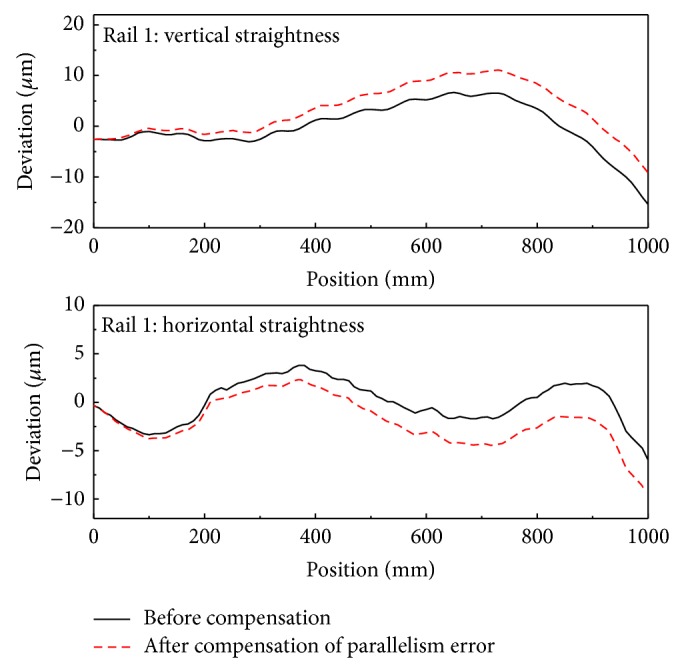
Straightness errors before and after compensating for parallelism errors.

**Figure 15:**
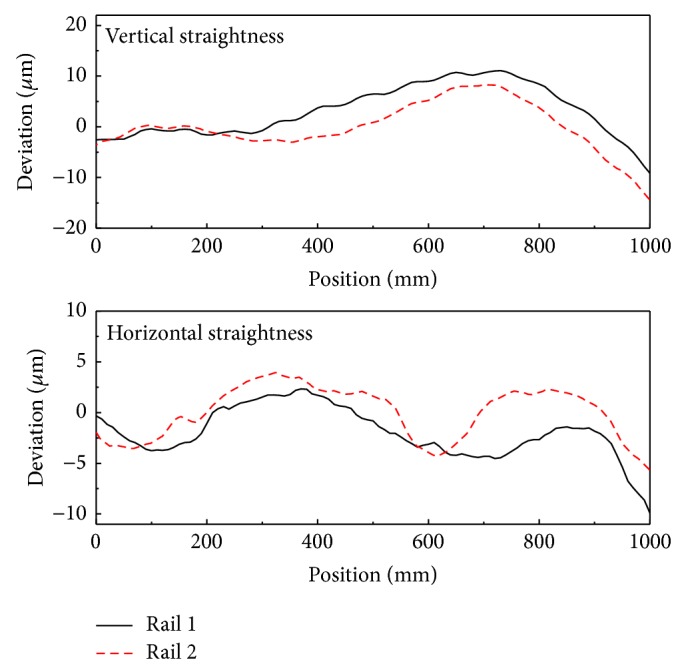
The straightness error of the bearing block for each rail.

Figure 14 shows the straightness error of the first rail obtained before and after compensation for parallelism error in the vertical and horizontal directions. For the vertical direction, the first rail is located toward positive *z*-direction against the second rail with increasing *x*; therefore, the roll error of the stage would be negative with *x* position. In the horizontal direction, the first rail was tilted negatively in the *y*-axis as *x* increased.  Figure 15 shows the straightness error of the bearing block for each rail in both the horizontal and vertical directions.

### 3.2. Experimental Verification and Discussion

**Figure 16:**
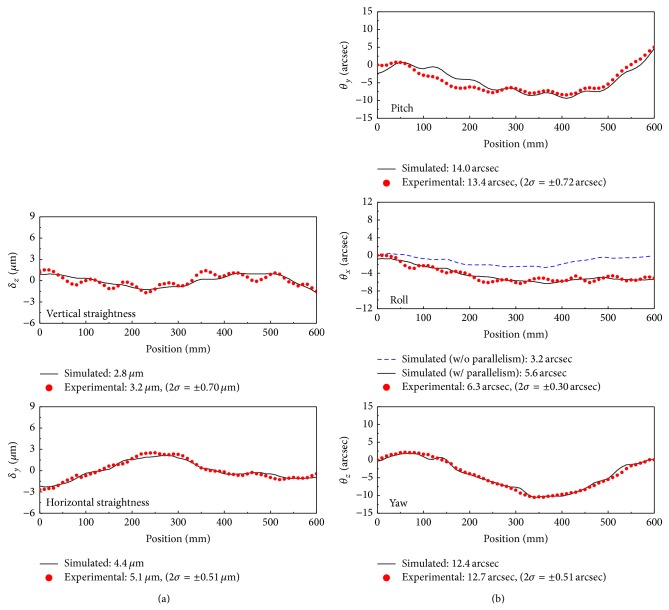
Comparison of calculated and measured data. (a) Linear components of the error and (b) the angular components.

Figure 16 shows a comparison of the calculated and measured motion errors in the linear motion bearing stage. The calculated motion errors were obtained by (12), using the measured straightness errors of the bearing block, as described in Section 3.1. The motion errors were measured using a laser interferometer and an electronic level meter on the table position at *z*_*c*_ = 81 mm. Data were averaged following five measurements. Although the horizontal straightness error of the stage included error due to the roll error of the stage, the influence on the horizontal straightness error was also calculated using (12).

Overall, the results show a good agreement, especially the horizontal straightness and yaw errors. However, some differences were observed in the vertical straightness, pitch, and roll errors. Sinusoidal components with a period of 80 mm were evident, as shown in Figure 16. We think that these errors were introduced as a result of the vertical rail-form errors, as the rails were fastened to the base using bolts that were separated by 80 mm, as shown in Figure 15. However, the causes of the small differences in the magnitude of the sinusoidal components between simulated and measured motion errors in the vertical direction are not clear, and so further investigation is required.

The calculated horizontal straightness and yaw errors were in good agreement with the measured data because of the absence of error sources, such as fastening bolts on the rails, as shown in Figure 16. The second graph of Figure 16(b) shows the effect of parallelism error in the calculation of the roll error; the measured roll error was in very good agreement with the calculated data.

## 4. Conclusion

We have described a method of calculating five components of the motion error in a linear motion bearing stage. The motion error was calculated using the reaction forces of bearings and equilibrium in the forces and moments on the stage. The method uses the reaction forces calculated based on the bearing stiffness and straightness errors of a bearing block, rather than rail-form errors, which are used in the transfer function method. It is not straightforward to obtain the rail-form errors in a linear motion bearing stage because of circular-profile grooves in the rails, whereas the straightness error of the bearing block can be obtained easily during the assembly process.

The reaction forces were calculated using the method described here and were compared with those calculated using the transfer function method. The horizontal straightness errors at a target point were compensated for to eliminate errors due to roll error, and parallelism errors between the two rails were taken into account. The motion error calculated using the method described here was in good agreement with experimentally measured data.

The method described here enables engineers to assess the allowable straightness error of a bearing block when assembling a stage, and to compensate for the error in the rails to improve the accuracy.

## Figures and Tables

**Table 1:** Specifications of the linear motion bearing stage.

Mechanical	Bearing type	Ball bearing (THK SHS30R)
	Number of bearings per rail, *m*	2
	Number of rails, *n*	2
	Distance between two rails, *l*_*y*_ (mm)	178
	Distance between two blocks, *l*_*x*_ (mm)	190
	Table length (mm)	300
	Rail length, *L* (mm)	1100
	Measurement length of straightness error of a bearing block, *L*′ (mm)	1000
	Pitch of assembly bolts on the rail (mm)	80
	Measurement point, *z*_*c*_ (mm)	81
	Distance between center of bearing block and Wollaston prism, *d*_*z*_ (mm)	51

Actuation	Actuator	310-4N (Trilogy)
	Scale	RGH24 (Renishaw)
	Controller	UMAC (DeltaTau)
	Drive	XSL230-36 (Copley)
